# Supplementary material for: Near-universal same-day linkage to ART care among newly diagnosed adults living with HIV: A cross-sectional study from primary health facilities, in urban Malawi
Source: PLOS Glob Public Health. 2023 Jun 16;3(6):e0001436. doi: 10.1371/journal.pgph.0001436 (PMC10275418; doi:10.1371/journal.pgph.0001436)
Supplement: S1 Checklist — (DOCX) [file pgph.0001436.s001.docx]

**Checklist for Same-day linkage to ART study**

Heath Centre: ________________________________________________________________________________________

Date of Inspection: ______________________________________________________________________________________

Location (e.g. General Assistant’s Area/Workroom/Art room): ____________________________________________________

| NO | OBSERVATION | RESPONSE | COMMENT |
| --- | --- | --- | --- |
| 1 | Number HTS counsellors at the facility |  |  |
| 2 | Number of ART providers at this facility |  |  |
| 3 | Number of experts’ clients at this facility |  |  |
| 4 | Duration (in hours) of expert clients service operation in a day |  |  |
| 5 | What time do the facility open? |  |  |
| 6 | Time facility closes |  |  |
| 7 | Number of shifts in a day. |  |  |
| 8 | Do expert client services offer during lunch hour |  |  |
| 9 | Average time taken with the client at HTC (pretest- test and post test) |  |  |
| 10 | Average time client take with the expert client (expert client counselling) |  |  |
| 11 | Average time client take at ART provider |  |  |
| 12 | Number of Counselling rooms in at this facility |  |  |
| 13 | Is privacy offered in expert client room |  |  |
| 14 | Is privacy offered in HTS rooms |  |  |
| 15 | Is privacy offered in ART rooms |  |  |
| 16 | Distance from HTC to ART |  |  |
| 17 | Availability of Navigators at this facility |  |  |
| 18 | Number of Navigators available at the facility |  |  |
| 19 | Number of times in the last quarter HIV testing materials have been out of stock. |  |  |
| 20 | Number of times ART drugs went out of stock at this facility in the last quarter |  |  |
| 21 | Number of times in a quarter the HTC counsellor was not available at the health facility |  |  |
| 22 | Number of times in a quarter, the expert client was not available to offer the service at the facility |  |  |
| 23 | Number of times in a quarter the ART provider was not available in to offer the service to the new positives |  |  |
