# Supplementary material for: Near-universal same-day linkage to ART care among newly diagnosed adults living with HIV: A cross-sectional study from primary health facilities, in urban Malawi
Source: PLOS Glob Public Health. 2023 Jun 16;3(6):e0001436. doi: 10.1371/journal.pgph.0001436 (PMC10275418; doi:10.1371/journal.pgph.0001436)
Supplement: S1 Questionnaire — (DOCX) [file pgph.0001436.s002.docx]

**Same- day linkage to ART Program of newly diagnosed HIV adults. Participant questionnaire**

**PID: Date:**

| **NO** | **QUESTIONS AND INSTRUCTIONS** | **RESPONSE** | **CODE** |
| --- | --- | --- | --- |
| **Respondent Socio-demographic characteristics** | |  |  |
| 1 | Time arrived at the clinic: ___: ____hrs. (24-hour clock)  Nthawi yofikira pachipatala: : Maola  (wotchi ya maola 24) |  |  |
| 2 | Time left clinic: ___:___hrs. (24 hr. clock)  .Nthawi yochokera pachipatala : :  (wotchi ya maola 24) |  |  |
| 3 | Where do you live?  Mumakhala kuti? |  |  |
| 4 | How did you travel from home to the clinic (mode of transport)?  Munayenda bwanji kuchokera kwanu kudzafika ku chipatala ( Njira ya mayende) | On foot  pansi | 1 |
|  |  | Public transport  Galimoto/ Njinga yolipila | 2 |
|  |  | Private transport  Pagalimoto/ Njinga yanu | 3 |
| 5 | Gender of the person interviewed | Female  Mkazi  If female  Pregnant | 1  Yes  No |
|  |  | Male  mwamuma | 2 |
| 6 | Age of the person interviewed or date of birth  Zaka za munthu amene akufunsidwa mafunso kapena Tsiku lobadwa | …………………………**years**  **……./……./…..**  **………………………………. Zaka**  **………../…………./………** |  |
| 7 | Highest level of education completed | No formal education  Sinapite ku sukulu ya mkalasi | 1 |
|  |  | Primary School  Sukulu ya pulayimale | 2 |
|  | Pa mapeto amaphunziro | Secondary School  Sekondare sukulu | 3 |
|  |  | Tertiary  Koleji | 4 |
|  |  | Adult literacy  Suluku ya kwacha | 5 |
| 8 | What is your religion? | Christian  chikhirisitu | 1 |
|  |  | Islam  chisilamu | 2 |
|  | Ndinu achipembedzo chanji? | African Traditional Religion  Chipembezo cha makolo | 3 |
|  |  | None  Palibe | 4 |
|  |  | Others (specify)…………………………………..  zina  Tchulani…………………. | 99 |
| 9 | Marital status  Ndinu okwatira kapena osakwatira? | Married  okwatira | 1 |
|  |  | Single/never married  osakwatira | 2 |
|  |  | Divorced  Banja linatha | 3 |
|  |  | Widowed  anamwalira | 4 |
|  |  | Others (specify)…………………………………..  Zina  Tchulani……….. | 99 |
| 10 | Number of sexual partners  Chiwerengero cha abwenzi ogonana nawo | ……………………………. |  |
| 11 | What is your occupation | Unemployed  Sindimagwira ntchito | 1 |
|  |  | Self employed  Ndili pantchito yodzilemba ndekha | 2 |
|  | Mumagwira ntchito yanji? | Employed  Ndili pantchito yolembedwa | 3 |
|  |  | Business  Bizinesi | 4 |
|  |  | Casual labour  maganyu | 5 |
|  |  | Skilled work  Ntchito za manja | 6 |
|  |  | Student | 7 |
| **Social Economic Status** | |  |  |
| 12 | Source of water | Borehole  mjigo | **1** |
|  | Gwelo la madzi | Tap water  Madzi apa mpopi | **2** |
|  |  | Public tap  Mpopi wa chitukuko | **3** |
|  |  | River / Stream  mtsinje | **4** |
| 13 | Source of lighting  Gwelo la kuwala | Electricity  magetsi | **1** |
|  |  | Lamp/ Candles  Nyali / kandulo | **2** |
|  |  | None  palibe | **3** |
| 14 | Type of toilet  Mtundu wa chimbudzi | Flush toilet  Cha madzi (chogejemura) | **1** |
|  |  | Pit latrine  chokumba | **2** |
|  |  | None  palibe | **3** |
| 15 | Main Mode of cooking used  Njirayodalilika imene mumagwilitsa nchito pophika | Electricity  Magetsi | **1** |
|  |  | Charcoal  makala | **2** |
|  |  | Firewood  nkhuni | **3** |
| 16 | Do you drink alcohol?  Mumamwa zolezeretsa? | Yes  Eya | **1** |
|  |  | No  ayi | **2** |
|  |  |  | **2** |
| 17 | Do you smoke?  Mumasuta? | Yes  Eya | **1** |
|  |  | No  ayi |  |
|  |  |  | **2** |
|  | **Knowledge and acceptance of HIV status**  **Kudziwa ndi kuvomereza za kachilombo ka HIV.** |  |  |
| 18 | Did you voluntarily undergo HIV test or were you requested by a health worker to have HIV test?  Munapita koyezetsa magazi mwakufuna kwanu kapena munachita kuwuzidwa ndi achipatala kuti muyezedwe magazi? | Voluntarily  Mwakufuna kwanga | 1 |
|  |  | Requested  ndinapemphedwa | 2 |
|  |  | Others (specify)……………………………  zina  (tchulani)…………………………………. | 99 |
| 19 | Did you fully accept the results of your test?  Kodi munavomereza kwathuthu zotsatira za zoyesa zanu? | Yes  eya | 1 |
|  |  | No  ayi | 2 |
| 20 | Did you receive counselling before and after HIV test at VCT?  Munalandira uphungu musanayezetse komanso mutayezetsa HIV koyezetsera magazi? | Yes  eya | 1 |
|  |  | No  ayi | 2 |
| 21 | How would you rate the pretest and posttest counselling you received from the HTC counsellor?  Kodi uphungu umene munalandira musanayedzetse komanso mutayezedwa magazi kuchokera kwa mphungu oyeza magazi mungaufotokoze bwanji/ unali otani? | Excellent  Wabwino kwambiri | 1 |
|  |  | Good  wabwino | 2 |
|  |  | Average  Wapakati kati | 3 |
|  |  |  |  |
|  |  | Bad  wosakhala bwino | 5 |
| 22 | How would you rate the counselling you received from the expert Client?  Kodi uphungu umene munalandira mutayezedwa magazi kuchokera kwa akatswiri mungaufotokoze bwanji/ unali otani? | Excellent  Wabwino kwambiri | 1 |
|  |  | Good  wabwino | 2 |
|  |  | Average  Wapakati kati | 3 |
|  |  |  |  |
|  |  | bad  wosakhala bwino | 5 |
| 23 | How can you describe the time you have been waiting to commence the service?  Mungafotokoze bwanji za nthawi imene munakhala mukuyembekeza kuti muyambe kulandira chithandizo | Less than 1 hour  Yosaposela ola limodzi | 1 |
|  |  | Between 1 hour and 2 hours  Pakatikati pa ola limodzi ndi ma ola awiri | 2 |
|  |  | Between 2 hours and 4 hours  Pakatikati pa maola awiri ndi maola folo | 3 |
|  |  | More than 4 hours  Kupyola maola 4 | 4 |
| 24 | How can you describe your interaction (relationship) with the HTC counselor during the process?  Mungafotokoze bwanji za ubale wanu ndi munthu opereka uphungu? | Excellent  Wabwino kwambiri | 1 |
|  |  | Good  wabwino | 2 |
|  |  | Average  Wapakati kati | 3 |
|  |  | Bad  Wosakhala bwino | 4 |
| 25 | How can describe your interaction (relationship) with the Expert Client counselor during this process?  Mungafokoze bwanji za machezedwe anu (ubale) ndi kadaulo wa uphungu. | Excellent  Wabwino kwambiri | 1 |
|  |  | Good  wabwino | 2 |
|  |  | Average  Wapakati kati | 3 |
|  |  | Bad  Wosakhala bwino | 4 |
| **Attitudes and perceptions** | |  |  |
| 26 | Are you ready to share the test results with your partner or family member?  Mwakonzeka kuwadziwitsa a chikondi anu kapena mmodzi wa achibale anu zotsatira za magazi anu? | Yes  eya | 1 |
|  |  | No  ayi | 2 |
| 27 | If answer for 26 is no probe for the reasons  Ngati yankho pa 26 ndi ayi funsitsitsani zifukwa zake. | ……………………………………………………… | |
| 28 | Do you think that you are going to be treated differently in the society because you are HIV positive?  mukuganiza kuti anthu a mdera lanu azakhalananu mosiyana ndi mmene amakuonerani chifukwa choti muli ndi ka chilombo ka HIV? | Yes  Eya | 1 |
|  |  | No  ayi | 2 |
| 29 | If answer to 28 is yes, in what way?  Ngati yankho ndi eya pa 28, munjira yanji? | ………………………………………………………… | |
| 30 | Do you know anyone who was found HIV positive and he/she is on ART?  Kodi mukudziwa aliyense amene anapezeka ndi kachilombo ka HIV ndipo pano akulandila mankhwala ama ARV? | Yes  Eya | 1 |
|  |  | No  Ayi | 2 |
| 31 | Are you ready to start ART today?  Ndinu okonzeka kuyamba ma ARV lero? | Yes  Eya 1 | |
|  |  | No  ayi 2 | |
| 32 | If yes to 31, state the reasons for starting ART  Ngati ndi eya pa 31, tchulani zifukwa zomwe mukuyambira ma ARV | Wants to be healthy 1  Ndufuna kukhala wathanzi | |
|  |  | Feeling Sick 2  ndikudwala | |
|  |  | Fear of Death 3  Ndikuopa kufa | |
|  |  | Reduces risk of HIV transmission to my partner 4  Kumachepetsa chiopysezo chopatsila kachilombo kwa amuna kapena akazi anga  To reduce my viral load 5  Kuchepetsa/ kutsitsa kuchuluka kwa tizilombo ta HIV mthupi mwanga.  Zina  (tchulani)……………… | |
| 33 | IF No to 32, state the reasons for not starting ART  Ngati ndi ayi pa 32, tchulani zifukwa zomwe simukuyambira ma ARV. |  | |
|  | | Fear of stigma  Kuopa kusalidwa | 1 |
|  | | Fear of side effects  Kuopa zoipa zama ARV | 2 |
|  | | Feeling healthy  Ndikuzimva wa thanzi | 3 |
|  | | Fear of status disclosure  Ndikuopa kuulura za mmene ndiliri | 4 |
|  | | Religious beliefs  Zikhulupiliro za mpingo | 5 |
|  | | Cultural beliefs  Zikhulupiliro za chikhalidwe | 6 |
|  | | Other  (specify)………………….  Zina  (tchulani)……………….. | 99 |
| **Institutional characteristics** | |  |  |
| 34 | Is this health facility accessible for you to receive services when needed?  Kodi chipatala chino ndichofikirika kwa inu pozalandirako chithandizo chikafunika? | Yes  Eya | 1 |
|  |  | No  ayi | 2 |
| 35 | Do you think there is privacy in accessibility of ART at this health facility?  Kodi mukuganiza kuti pali chisinsi pa kalandiridwe kama ARV pa chipatala pano? | Yes  Eya | 1 |
|  |  | No  ayi | 2 |
| 36 | Do you find any problem with this health facility in terms of services which are provided?  Kodi mukupezapo vuto lirilonse ndi chipatala chino pa zithandizo zomwe zikuperekedwa? | Yes  Eya | 1 |
|  |  | No  ayi | 2 |
| 37 | If the answer for 36, is yes the probe for the problems.  Ngati yankho pa 36 ndi eya, funsitsitsani zovutazo. | ……………………………….. | 99 |
| 38 | Do you find the distance between the VCT and ART clinic is favorable to you?  Kodi katalikidwe pakati pa koyezera magazi ndi kuchipatala cha ma ARV ndi za bwino kwa inu? | Yes  Eya | 1 |
|  |  | NO  ayi | 2 |
| 39 | Do you find the location of Expert client in the VCT clinic Helpful / favorable to you?  Kodi malo omwe kadaulo wa uphungu akupezeka kumalo oyezera magazi ndi a bwino kwa inu? | Yes  Eya | 1 |
|  |  | No  ayi | 2 |
| 40 | Did you find any Navigation assistance at this clinic?  Kodi munapeza aliyense okulondolerani pa chipatala pano? | Yes  Eya | 1 |
|  |  | No  ayi | 2 |
| 41 | If yes to 40, did you find it helpful  Ngati ndi eya pa 40, munaziona kuti ndizothandiza? | Yes  eya | 1 |
|  |  | No  ayi | 2 |
| 42 | If no to 40, probe the reason  Ngati ndi ayi pa 40, funsitsitsani chifukwa chake. | ….. |  |
| END |  | …………………………………………………………. | |
