# Supplementary material for: Near-universal same-day linkage to ART care among newly diagnosed adults living with HIV: A cross-sectional study from primary health facilities, in urban Malawi
Source: PLOS Glob Public Health. 2023 Jun 16;3(6):e0001436. doi: 10.1371/journal.pgph.0001436 (PMC10275418; doi:10.1371/journal.pgph.0001436)
Supplement: Attachment — Submitted filename: Cover letter Responses to Reviewers Plos final PLOS.docx [file pgph.0001436.s003.docx]

2 Feb 2023

Editor: PLOS GLOBAL PUBLIC HEALTH

Dear Editor,

On behalf of my co-authors, we thank you for your valuable comments and the opportunity to resubmit a revised draft of our manuscript of a research article entitled “**Near-universal same day linkage to ART care among newly diagnosed adults living with HIV: A cross sectional study from primary health facilities, in urban Malawi**” for exclusive consideration for publication in PLOS Global Public Health.

We thank the reviewers for their thorough review and believe that the manuscript has been significantly improved with their input.

Sincerely,

Please find our responses in red to the reviewer comments below. Line numbers refer to the manuscript with tracked changes.

Reviewer # 1

1.It is not clear whether the working title is one at the page 1 or at the page 3 (Line 49 and 50)

Thank you for your comment. The working title is the one on page 3 under line 52 and it is “Same-day linkage to ART in Malawi”

Introduction
1. Introduction could be structure better with each paragraph consistently summarizing one main idea, for example, the first paragraph could focus on epidemiology profile globally, regionally and specifically with Malawi. It could follow with some policy context around these numbers. (e.g., it is unclear what are data that limited in line 61, and the goal of 95-95-95 that had not been introduced properly in the line 75 page 4).

We have made changes to the introductions section in lines 58- 60 and have included some epidemiological profile of HIV at global level. We have clarified in lines 86-87 that the limited data is on same day ART prevalence and its associated factors in Malawi. Malawi’s UNAIDS second goal was to have 95% of those that are HIV positive on sustained ART by 2030. This has now been properly introduced in lines 102-104.

2. The next (third paragraph) could focus on any past studies on the factors (the individual-, facility is- and system-level factors) that influences uptake of same-day linkage to ART initiation in Malawi and SSA. Currently it is a little bit lacking, with more focused are given only to the availability of EC (expert clients)

We have revised the paragraphs as requested. The second paragraph (lines 70 - 87) now includes findings from past studies on linkage to ART in Malawi and the region. However, these studies were not on **same day** linkage. There is paucity on evidence from studies conducted in this region of Africa on same day linkage prevalence and associated factors. This section has included only one study from Ethiopia on same day linkage and its associated factors.

3. The knowledge gap and the primary aim of this paper are a bit vague in the last paragraph of this introduction.

More information has been provided in the second paragraph in lines 70-87 and together with information on lines 110-113 in the third paragraph, we emphasize the knowledge gap on factors which promote same day ART initiation in Malawi and other countries in sub-Saharan Africa.

I will suggest authors to also consistently use the terms throughout the paper (e.g., health care centre or health facility)

The term health facility has now been consistently used in the paper.

**Methods**
1. As I mentioned in the overall comment, this paper lacks a framework to describe the different level of individual-, health facility, and system- level factors that influence the uptake of same-day ART initiation.

Thank you for your comment. In the Methods section, under lines 158 – 165, we have provided information on the variables that were collected under the personal, health facility and health systems categories.

2. Sample size: I do not understand on the justification of the selection of Blantyre district (other than what been mentioned in Introduction about higher coverage of same-day ART), the context of this district, the size and justification of selection of only two facilities (south Lunzu and Limbe) and if there is randomization process taken place in this selection (or all are selected purposefully and how this maybe create bias).

Blantyre district has the highest HIV prevalence of 14.2% and therefore the need to investigate determinants that will encourage same day ART initiation is high. The health facilities are busy ones and were selected for convenience inorder to achieve the targeted sample size easily. South Lunzu health facility represented rural health facilities while Limbe represented semi urban ones. This was not conducted in all health facilities because it was a pilot study. This information and that of the context and size of Blantyre district has been provided in the Introduction section in line 61 of the Introduction section and Methods section in lines 120 – 125.

3. There is no detailed explanation about variables (other than the main outcome) – such as predictors, potential confounders or other type of variables.

The dependent variables that fell under personal, health facility and health systems categories have been described in the Methods section in lines 158 - 165.We ensured to collect a lot of variables as much as possible inorder to ensure that data on possible confounders are also collected.

Data sources are not clearly defined and questionnaire that been used in this study are not attached. While also there is no explanation how the questionnaire is made, and whether it has been piloted before.

A structured questionnaire was used on the newly diagnosed people living with HIV (PLHIV) and checklist was used on health care workers (HCW) who were leaders of each health facility. These data collection tools were put together specifically for this study and were piloted at a different health facility before the study commenced. This led to revision of some questions in the questionnaire, before it was implemented on the study. This information has been described in lines 150 - 159.

5. This paper will very be benefitting with a statistical method that can check any association between main outcome and variables of interest. At this current state, the analysis seems lacking.

Thank you for your comment on this. We were unable to run statistical methods to check associations on this paper because only 2% of study participants did not start same day ART.

**Results**
1. Inconsistent way to present the results, line 132 page 6, also line 152 – 153

The results have been presented as mean age (standard deviation). Please refer to lines 185 – 187 and 209– 211.

2. Table 1. Source of water – tap, the number is mistype

This has been corrected

3. Line 155, health facility leaders are not defined (or explained) before

These have been defined in line 140 under the methods section.

4. The terminology “views of health care workers and clients” in the page 153 seems misleading since it seems like a qualitative study instead.

Thank you for the observation. The word ‘views’ has been changed to ‘responses’. Please refer to line 212.

**Reviewer #2:** Article well-written.
1. Topic: Topic followed scientific format
2. Abstract: Abstract well written
3. Introduction section
a. In line 52, author did not define UNAIDS at the initial, knowing that USAID is an acronym.

Thank you for the comment, this has been defined in line 58.

b. In line 53, 54 and 55, Author can reverse by joining a two sentence to a singular one for example; In Malawi, HIV prevalence among adults aged 15- 49 years is
8%, and 97.9% of PLHIV are taking antiretroviral therapy (ART) with the highest ((14.2%) occurring in Blantyre (2).

The sentences have been joined and can be found in lines 60,61 and 62.

4. Methods: Methods described the scientific approach the manuscript.

5. Results: Results were well written however, the manner of table presentation should comply with the scientific format of presenting results in a tabular form. For example, table should contain 3 lines; top, middle and the bottom lines.

Thank you for your comment, the authors have followed the PLOS Global Public Health journal guidelines for tables.

Rachel Chihana Kawalazira (MBBS, MSc)

Johns Hopkins Research Project

Blantyre, Malawi

rkawalazira@jhp.mw
